# Applications of Artificial Intelligence in Ophthalmology: Glaucoma, Cornea, and Oculoplastics

**DOI:** 10.7759/cureus.73522

**Published:** 2024-11-12

**Authors:** Kristie M Labib, Haider Ghumman, Samyak Jain, John S Jarstad

**Affiliations:** 1 Department of Ophthalmology, University of South Florida Morsani College of Medicine, Tampa, USA

**Keywords:** artificial intelligence in medicine, cornea, glaucoma, oculoplastics, ophthalmology

## Abstract

Artificial intelligence (AI) is transforming ophthalmology by leveraging machine learning (ML) and deep learning (DL) techniques, particularly artificial neural networks (ANN) and convolutional neural networks (CNN) to mimic human brain functions and enhance accuracy through data exposure. These AI systems are particularly effective in analyzing ophthalmic images for early disease detection, improving diagnostic precision, streamlining clinical workflows, and ultimately enhancing patient outcomes. This study aims to explore the specific applications and impact of AI in the fields of glaucoma, corneal diseases, and oculoplastics. This study reviews current AI technologies in ophthalmology, examining the implementation of ML and DL techniques. It evaluates AI's role in early disease detection, diagnostic accuracy, clinical workflow enhancement, and patient outcomes. AI has significantly advanced the early detection and management of various ocular conditions. In glaucoma, AI systems provide standardized, rapid identification of disease characteristics, reducing intra- and interobserver bias and workload. For corneal diseases, AI tools enhance diagnostic methods for conditions such as keratitis and keratoconus, improving early detection and treatment planning. In oculoplastics, AI assists in the diagnosis and monitoring of eyelid and orbital diseases, facilitating precise surgical planning and postoperative management. The integration of AI in ophthalmology has revolutionized eye care by enhancing diagnostic precision, streamlining clinical workflows, and improving patient outcomes. As AI technologies continue to evolve, their applications in ophthalmology are expected to expand, offering innovative solutions for the diagnosis, monitoring, treatment, and surgical outcomes of various eye conditions.

## Introduction and background

Artificial intelligence (AI) is increasingly influencing the field of ophthalmology, revolutionizing eye care with various virtual models designed to analyze and organize medical data. These systems, employing machine learning or deep learning techniques with artificial (ANN) or convolutional neural networks (CNN), mimic human brain functions and improve accuracy through data exposure [1]. AI learning processes include unsupervised learning (recognizing patterns), supervised learning (identifying pre-labeled characteristics), and reinforcement learning (learning through rewards or penalties). Notably, AI has significantly advanced the early detection and management of various ocular conditions. AI-powered tools analyze ophthalmic images to identify diseases at their earliest stages, enhancing treatment outcomes and slowing disease progression. These advancements enhance diagnostic accuracy, streamline clinical workflows, and improve patient outcomes. As AI evolves, its integration into ophthalmology is expected to expand, bringing innovations pertaining to diagnosis, monitoring, treatment, and surgical outcomes.

The creation of advanced artificial intelligence technologies relies on the central development of neural networks. Similar to the neurological system seen in organisms, the neural networks that develop AI systems operate through layered units that mimic neurons. These neurons are able to complete tasks systematically and functionally with the added benefit of developed intelligence [2]. CNNs employ layered and multi-faceted neural networks for specific purposes, the most prevalent being image analysis in ophthalmology. These networks have the capability to better analyze healthcare-related and ophthalmology-related factors, leading to more developed outcomes. CNNs are also able to employ necessary image analysis tools including segmentation and detection for the purposes of analysis [3]. ANNs play a significant role in the development of technological intelligence and the ability of technology to think in a manner similar to human capabilities. ANNs are key to technological integration and development in areas across ophthalmology [4]. While ANN relies on text or tabular data to solve complex problems, CNN relies on image and video inputs to discern meaningful information. Because of its associated inputs and ability to meaningfully identify images, CNN can provide more utility in ophthalmology.

Similarly, distinctions can be seen between forms of AI, with the most used being machine and deep learning. Machine learning (ML) provides the essential necessities for the composition of AI. With the ability to recognize patterns and build on them, ML allows for a great degree of technological independence [5]. Human intelligence is required to complete statistical models, which is a necessity that can be replaced by the involvement of ML. Furthermore, ML is able to refine its understanding through the developed patterns, increasing its output speed and accuracy [5]. Within ophthalmology, ML is able to note significant conclusions from large sets of data that can be used to further the patient care process [5]. Deep learning (DL) utilizes multiple layers of neuronal networks to further develop intelligence-based capabilities. These layers are able to further detect and utilize derived features for constant improvement [6]. The current integration of DL into ophthalmology has resulted in improved capabilities and powerful pattern detection, which shows promise for increased utility in the future [6].

**Figure 1:**
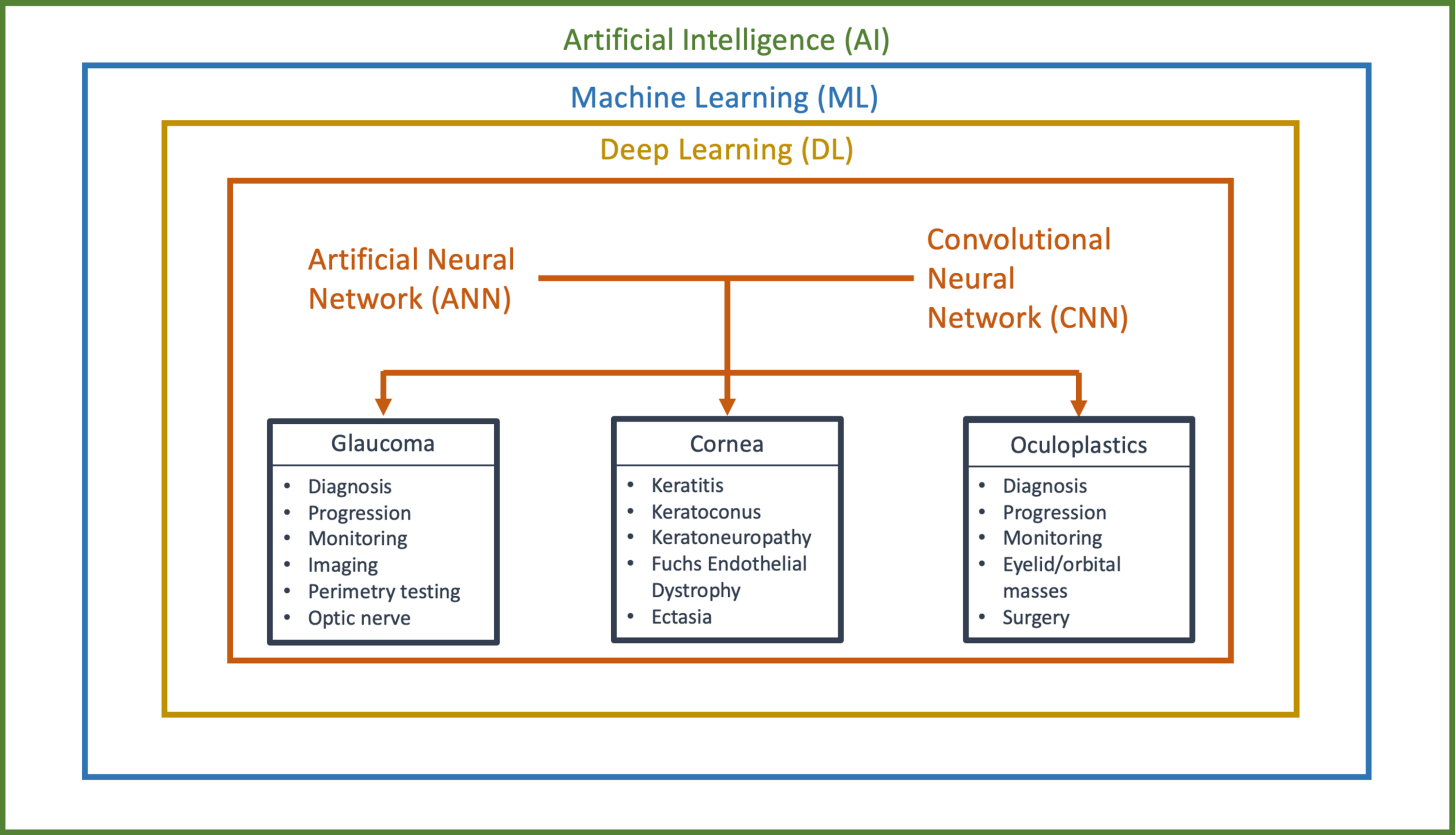
Components of Artificial Intelligence and Applications

The adoption of AI in various medical disciplines is accelerating, with ophthalmology standing out as particularly apt for AI integration. This suitability stems from its extensive reliance on imaging and data for activities such as screening, diagnosing, prognosticating, and treating eye conditions. The wide array of imaging techniques utilized in ophthalmology offers abundant opportunities for developing AI programs using this imaging data. AI's role in ophthalmology is revolutionary, holding the potential to significantly enhance diagnostic precision, facilitate early disease detection, and elevate the quality of patient care. Several recently developed AI models have proven their effectiveness in ophthalmological care, highlighting the transformative impact of AI in this domain. With these advancements, AI is anticipated to play an essential role in the future of ophthalmology, revolutionizing the management of eye disorders. This study aims to examine the applications of AI in ophthalmology, specifically focusing on glaucoma, cornea, and oculoplastics (Figure 1).

## Review

Artificial intelligence and glaucoma

AI and Glaucoma Diagnosis

Glaucoma is the second leading cause of blindness worldwide, with blindness mostly being irreversible [7]. As of 2020, an estimated 57.5 million people were affected by glaucoma worldwide, with this number expected to reach 111.8 million by 2040 [7]. Studies have found that 10-33% of people with glaucoma present with advanced disease and visual impairment due to the asymptomatic nature of glaucoma often found early in the disease process [8-10]. Moreover, improved glaucoma screening modalities are being developed for early detection and intervention prior to irreversible vision loss.

The diagnosis of glaucoma often depends on the combination of multiple diagnostic tools, including optical coherence technology (OCT) imaging, visual field testing with standard automated perimetry assessment (SAP), fundoscopic imaging, and clinical assessment. AI has been found to be a valuable tool in glaucoma diagnosis by providing standardized and rapid identification of disease characteristics while simultaneously limiting intra- and interobserver bias. AI further reduces the workload of image interpretation for glaucoma experts with high accuracy (area under receiver operating curve (AUC) values greater than 0.90 in various structural and functional glaucoma testing modalities) [11].

AI systems are found to provide enhanced structural differentiation between normal and glaucomatous eyes. When machine learning classifiers are trained, they can identify relevant optic disc topographic parameters, which can therefore highlight glaucomatous eye patterns. Topographic parameters include peak height contour, disc area, and cup shape [12]. Furthermore, ANNs can aid in the segmentation and classification of glaucoma biomarkers in fundus color photography. Therefore, AI systems quantify cup-to-disc ratios and detect glaucoma-related features aiding physicians in glaucoma diagnosis and management [13,14]. MacCormick et al. found that a spatial algorithm was accurate in its detection of glaucomatous (AUC = 0.996) and healthy discs (AUC = 0.91) [13]. Similarly, Ting et al. validated a deep learning system that was able to detect possible glaucoma with an AUC of 0.942, sensitivity of 96.4%, and specificity of 87.2% [14]. In addition, trained DL networks can accurately stage glaucoma by measuring the optic nerve head volume and retinal nerve fiber layer thickness on OCT images and utilizing them as parameters for staging, as shown by an AUC of 0.962 [11,15]. There has been evidence that combining structural eye parameters (such as cup-to-disc ratio) and functional eye parameters (visual field measurements) are effective in identifying glaucoma with a sensitivity of 90% and specificity of 84% [16]. AI plays a promising role in improving glaucoma management by offering efficient and precise tools for glaucoma diagnosis.

AI and Glaucoma Progression

Glaucoma management is a complex process demanding clinician expertise, and multiple repeated tests over an extended period are required to assess glaucoma progression [17]. Fortunately, AI systems are being developed to help identify functional damage and structural damage as well as prognosis in glaucoma patients.

Artificial neural networks (ANNs) have been developed to discern visual field defects in glaucomatous eyes from standard automated perimetry data (SAP). A notable AI system to do this was developed in 1994, which was able to detect visual field defects proficiency almost at the level of trained readers, with 74% agreement. Subsequently developed AI systems have been able to perform as well or outperform glaucoma experts [18,19].

The progression of glaucoma differs between each patient, making it difficult to establish a standardized method for determining glaucoma prognosis. Fortunately, AI systems have demonstrated promising capabilities in assessing glaucoma prognosis, which can assist clinicians with decision-making. A study by Wen et al. found that deep learning networks can be trained to recognize spatial-temporal changes in Humphrey visual field (HVF) data and predict HVF results up to 5.5 years earlier with just one baseline HVF result as input [20]. Therefore, understanding the rate or extent of vision loss can help clinicians in determining the course of treatment for glaucoma patients to suggest whether more aggressive or conservative treatment is required. Machine learning systems may also be trained to identify abnormalities in visual fields among patients with ocular hypertension [21]. It was also found that in patients with ocular hypertension and normal visual field results, certain machine learning systems can identify abnormal visual fields an average of 3.92 ± 0.55 years earlier than conventional methods [21]. Being able to obtain an enhanced understanding of a patient’s glaucoma progression not only can assist in treatment planning, but it can also provide more information to patients about their disease.

Artificial intelligence and the cornea

AI in Keratitis

AI offers potential to be an effective tool in diagnosis and management of corneal diseases. With diseases such as keratitis, a prompt and accurate diagnosis is prudent to decrease the risk of antibiotic resistance and vision loss [22]. As a result, AI may assist clinicians with keratitis management by offering more accurate diagnostic methods than those in standard clinical practice [23]. Developed AI algorithms have been trained to identify pathological features associated with microbial keratitis, such as stromal infiltration, hyphema, leukocyte boundaries, and corneal edema boundaries [24]. Additionally, a 2003 study discussed a developed ANN that exhibited exceptional results when diagnosing corneal ulcers. The ANN was able to demonstrate 76% specificity in diagnosing bacterial keratitis and 100% specificity in fungal keratitis on 106 patients with infectious corneal ulcers [25]. The ability to develop ANNs with high specificity displays the vast potential for clinical implementation of AI for keratitis diagnosis.

AI in Keratoconus

AI may assist in the early diagnosis of keratoconus, which is beneficial as untreated keratoconus may cause irreversible blindness [26]. AI algorithms may supplement standard diagnostic methods to enhance the accuracy of clinical diagnosis. For instance, an AI algorithm was able to distinguish eyes as normal or developing keratoconus with a 94.1% sensitivity and a 97.6% specificity from corneal topography data [27].

Scheimpflug cameras may capture characteristics of the anterior and posterior cornea, and they are incorporated in AI algorithms created for keratoconus diagnosis. In one study that utilized these cameras, it was found that there was improved detection of subclinical keratoconus through the combination of ultra-high-resolution optical coherence topography and Scheimflug cameras in 121 eyes (AUC = 0.93) [28]. Furthermore, AI programs work like human brains as they synthesize findings from multiple forms of imaging to help identify pathology. An example of this was found in a recent study, where the Scheimpflug camera obtained four color-coded corneal topographic maps, which are the axial map, anterior and posterior elevation map, and pachymetry map [29]. The study found that the developed CNN was able to identify keratoconus with higher accuracy when utilizing all four topographic maps collectively than by utilizing only one type of map, with an accuracy rate of 0.9785 [29]. With AI programs approaching the functionality of human brains, there appears to be substantial potential of AI in the diagnosis and management of keratoconus.

AI in Diabetic Keratoneuropathy

AI algorithms may play a role in the early detection of diabetic keratoneuropathy, preventing irreversible corneal damage. In vivo confocal microscopy can assist with the assessment of corneal microstructure to help identify the extent of keratoneuropathy. As a result, trained CNN networks utilize confocal microscopic images to identify diabetic changes in corneal nerves and estimate severity based on measured nerve parameters [30-32]. Williams et al. developed a deep learning algorithm for the diagnosis of diabetic neuropathy with an AUC of 0.83, sensitivity of 68%, and specificity of 87% [31]. However, Meng et al. developed an improved algorithm reporting an AUC of 0.95, sensitivity of 91%, and specificity of 93% [32]. Therefore, AI tools may be a beneficial tool in the diagnosis of diabetic keratoneuropathy but may require further development to increase precision and accuracy.

AI in Fuchs Endothelial Dystrophy

AI tools have shown potential in the diagnosis of Fuchs endothelial corneal dystrophy both in its early and late stages. In a 2020 study, a deep learning classification network was trained by 18,720 anterior segment OCT images, which consisted of 9,180 images of healthy eyes, 5,400 images of early-stage Fuchs endothelial corneal dystrophy, and 4,140 images of late-stage Fuchs endothelial dystrophy [33]. This model produced exceptional results, obtaining a 97% specificity and 91% sensitivity in the detection of early-stage Fuchs endothelial corneal dystrophy, and a 92% specificity and up to 100% sensitivity in the detection of late-stage Fuchs endothelial corneal dystrophy. It also had a 98% specificity and a 99% sensitivity in distinguishing between any stage of Fuchs endothelial corneal dystrophy and normal eyes [33].

This finding is a breakthrough in the research area of Fuchs endothelial corneal dystrophy, as Fuchs is traditionally diagnosed by slit lamp examination, specular microscopy, corneal thickness measurement, and confocal microscopy. These tools are unable to visualize disease progression or track chronological changes in endothelium [33]. Therefore, AI technology may automatically classify images into the respective stage of the Fuchs disease process rather than relying on analysis to be performed by clinicians.

AI and Refractive Surgery Postoperative Complications

In the realm of refractive surgery, studies are investigating AI-driven methods of screening for ectasia, a rare but feared complication of refractive surgery. Ectasia is characterized as a progressive increase in myopia with steepening of the cornea and possible astigmatism that can largely reduce visual acuity [34]. The limitation of current diagnostic modalities is that they are unable to monitor chronological changes of ectasia or predict its prognosis [34]. Therefore, the role of AI in this disease process is to potentially enhance the utility of imaging in corneal ectasia management. Patients may be more predisposed to developing postoperative ectasia due to subclinical keratoconus or other causes of corneal mechanical weakness. Therefore, AI-driven screening methods that identify subtle anatomic abnormalities are being developed. Xie, et al. introduced a tomography-based screening system to assess the risk of developing postoperative ectasia, which was able to identify early keratoconus with a sensitivity of 90% and distinguish eyes that are suspect for ectasia with a sensitivity of 80% [35]. Therefore, AI can assist ophthalmologists in identifying eyes that are at risk for developing ectasia, where the eyes at risk can be followed closely to prevent or slow down the disease.

Artificial intelligence and oculoplastics

Role of AI in Oculoplastics

Eyelid/orbital pathology can both impact vision and have a profound impact on the cosmetic facial appearance of individuals [36]. As a result, offering precise methods of diagnosis and treatment may assist with providing the best possible outcomes for patients. AI has played a prominent role in assisting with disease identification by training network models to analyze large datasets [36]. AI can additionally assist in capturing accurate eye measurements to aid in surgical treatment planning for surgical management of eyelid and orbital disease. AI can also aid in postoperative management such as capturing images and identifying possible abnormalities that may lead to complications [36].

CNNs are among the more commonly implemented AI tools in eyelid and orbital disease management [36]. Features of an image are condensed and organized to create outputs that will ultimately allow for feature identification and segmentation. After sufficient training of CNN systems with computed tomography (CT) and magnetic resonance imaging (MRI) images, they may be used to identify abnormal tissue or pathology in orbital disease which can help identify abscesses, cellulitis, or abnormal enlargement of septal fat [36].

AI in Diagnosis and Monitoring of Eyelid and Orbital Disease

AI has played a role in the diagnosis of eyelid and orbital disease. Fu et al. discussed a successful CNN system that allowed for the segmentation of orbital abscesses in CT images of patients with cellulitis, which performed similar to a human expert [37]. AI has also been used to automatically diagnose orbital disease. For example, Song et al. proposed an AI algorithm to identify thyroid ophthalmoplegia on CT, which proved to be effective when utilized in clinical practice [38]. A developed CNN was able to effectively screen for ptosis with higher sensitivity (92%) than that achieved by non-ophthalmic general practitioners. Therefore, AI models may potentially serve as an effective screening tool in primary care settings. In addition, DL networks are able to diagnose both obstructive and atrophic meibomian gland dysfunction and differentiate them between healthy eyes with high diagnostic accuracy, ranging from 97% to 99% [39].

AI programs trained by MRI images can stage thyroid-associated ophthalmopathy (TAO), a condition requiring diagnosis and management to prevent vision loss. AI algorithms can identify edematous extraocular tissue to stage TAO with an accuracy of 86.3%, sensitivity of 75%, and specificity of 89.6% [40]. The advantage of utilizing AI for TAO staging is that it ensures consistent and objective identification of extraocular tissue edema, which can providers with less experience. In addition, dysthyroid optic neuropathy (DON) is a feared complication of TAO, as it may cause irreversible blindness with late detection. TAO may not present until its late stages, during which treatment options are limited. As a result, Wu et al. developed a radiomics nomogram model (AUC = 0.92), which was able to identify DON in patients with TAO more effectively than would be done with standard MRI (AUC = 0.80) [41]. As a result, AI models may detect DON early and be utilized to prevent irreversible vision loss.

AI and Diagnosis of Eyelid and Orbital Masses

AI has been found to effectively diagnose eyelid and orbital masses [42]. Han et al. discussed multiple ML networks that performed a qualitative classification of orbital cavernous venous malformations, one of the most common orbital masses diagnosed [42]. They can rapidly diagnose orbital cavernous venous malformations using non-enhanced CT images without requiring MRI or any contrast studies (AUC = 0.9448, accuracy = 88.06%), which may be helpful in those with contraindications to contrast agents.

Additionally, AI has the capacity to differentiate between ocular masses that are difficult to distinguish on images, which is essential as the identification of masses will determine surgical management options. Extensive training of DL networks may be utilized to identify subtle differences between multiple types of similarly appearing tumors. Bi et al. developed an AI system that was able to successfully differentiate between cavernous hemangiomas and schwannomas across multiple centers (accuracy = 91.13%) [43]. Xie et al. utilized radionomics along with imaging and clinical information to conduct a DL-based analysis that differentiates between ocular adnexal lymphoma and idiopathic orbital inflammation (AUC = 0.953), which are two types of orbital lymphoproliferative disorders that require very different treatment [44].

AI-generated models may also enhance and streamline cancer diagnosis. Deep learning-based CNNs have displayed promising results in differentiating benign lesions from cancer with AUC ranging from 0.908 to 0.950 [45]. A region-based DL AI system was even able to effectively differentiate benign and malignant eyelid lesions with similar accuracy to a senior ophthalmologist with AUC ranging from 0.899 to 0.955 [46]. These advancements highlight the potential of AI in the management of various eyelid and orbital lesions.

AI and Oculoplastics Surgery

AI may assist in obtaining accurate measurements of eyelid morphologic parameters prior to eyelid surgery. In traditional methods of obtaining eyelid measurements, values may be affected due to facial movements or changing facial expressions. As a result, AI may obtain automatic and objective measurements, which therefore may improve the accuracy of eyelid surgery. Some measurements that may be obtained using AI include the marginal reflex distance (MRD) in static and dynamic ocular photographs [47], pupillary distance, eye area, and average eyebrow height [48]. AI may also measure levator muscle strength, lid fissure height, and limbal reflex distance, which are essential for surgical treatment planning of ptosis [49].

AI has also been found to aid in eye cosmetic surgery, where a CNN-based eye model was created to aid oculoplastic surgeons in surgical planning [50]. Qu et al. found that patients who had their cosmetic operations planned by the CNN eye model achieved superior cosmetic results than those in the control group that were evaluated only by doctor experience [50]. The improved cosmetic results included a lesser degree of eye pouches, lower eyelid skin wrinkling, skin gloss, and enhancements in the lacrimal sulcus.

Limitations of artificial intelligence and its impact on jobs

Ethical Dilemma

Although artificial intelligence is progressing in the realm of ophthalmic disease diagnosis, screening, and treatment planning, there are some concerns that limit the extent of AI utilization in ophthalmic care [51]. Questions have been raised about the possible ethical concerns with AI [52], as errors in AI algorithms may be detrimental to patient care. AI programs may result in medical complications across racial groups and economic classes, causing a lack of health equity due to the lack of generalizability of AI systems [53]. This problem is stemmed from the concept of “overfitting”, meaning AI programs appear to be more accurate than they are in reality, as AI programs are usually trained from smaller data sets [52]. An additional ethical consideration of AI is its impact on the doctor-patient relationship, essential for delivering optimal patient care [51]. It was found that both patients and ophthalmologists have concerns about AI impacting doctor-patient relationships and hindering collaborative decision-making and the informed consent process [54].

The solutions to ethical concerns of AI can be found by improving the development strategy of AI algorithms and ensuring AI programs are applicable to specific target populations [51]. AI programs trained with large data sets for specific ophthalmology-related purposes will increase AI program accuracy and broaden target populations for the AI program [52]. Ensuring adequate oversight and legislation of AI programs by Food and Drug Administration (FDA) approval and close monitoring of programs may also address the ethical limitations of AI [51]. Consequently, AI would be closely surveilled to ensure it, maintaining national and international standards, which therefore would increase trust in AI programs [52,55]. Creating frameworks for AI implementation utilizing interdisciplinary support can assist in ensuring AI systems are utilized ethically and practically [51]. Philosophers, ethicists, and other physicians may collaborate to create AI application frameworks [53]. Although questions regarding ethics still remain, AI is evolving rapidly and continuing to improve to address and minimize ethical concerns [54].

Loss of Privacy

With the increasing usage of AI comes the concern about loss of privacy, as patient information is utilized to develop AI models [51]. There remains to be a question of which patient information is necessary for patient health and which needs to be kept in patient privacy, and this gray area must be delineated prior to using patient information to develop AI programs [56]. The concern about AI causing loss of privacy mainly stems from general mistrust in technology due to the current widespread use of malware, resulting in hacks and leaks of personal information [57]. Some of the more recently developed AI programs have been incorporating even more patient information, such as facial identity, but this poses a higher risk for security breaches causing loss of privacy [58].

The solutions to address concerns for loss of privacy can be found through regulations of AI programs, namely through the Health Insurance Portability and Accountability Act (HIPAA) [51]. HIPAA directly serves to protect patient information and specifically delineate which patient information will be utilized. It will ensure that patient information is being used strictly for patient health and that there are specific methods to ensure patient data is secure [51]. Each state itself additionally has protections to monitor and increase privacy for patients [56,59].

Data Set Development

AI algorithm accuracy is directly related to the quality of both the AI algorithm training and the specific data set that was used to train the AI algorithm [51]. AI algorithms perform more superiorly with data sets of higher quality and quantity [60]. Unsatisfactory patient data sets reduce AI algorithm accuracy, which would therefore impact patient care if applied in practice [51]. Data sets with unspecified or missing data result in overall a small set of usable data for AI program training, which would reduce confidence in the AI algorithm itself [61]. In addition, the cost associated with data set production has created an additional barrier to developing optimal data sets [51]. Despite having enough images from patients that may be used for AI algorithm training, images may be incompatible with the AI software due to image size or file style [62]. In addition, some images may lack labels or contain incorrectly labeled images, which hinders the process of training certain networks [51]. Therefore, optimal data sets are necessary for accurate AI algorithm development, but there are difficulties in bringing collected data into a compatible form for AI programs [62].

To address concerns dealing with the quality of data sets, solutions including synthetic data set development, data augmentation, and down sampling, which can help polish data sets to rectify AI program training [51,60]. The research side of ophthalmology can also largely benefit from the development of well-organized data sets [51]. AI holds the potential to unlock major advances in research, leading to major development in medical and ophthalmology-based understanding [51]. The use of large-scale computing can help organize data while limiting the potential for human error, allowing AI to derive clear and accurate results [60].

Impact on Jobs

Effective patient care is delivered when a physician demonstrates knowledge, empathy, compassion, and adaptability. As AI is changing substantially, some AI models have been found to demonstrate these qualities [63]. However, a report found that while 47% of jobs are at risk for automation, the risk for physicians and surgeons is a mere 0.4% [63]. Although some AI programs have demonstrated stellar diagnostic abilities during testing, some superior to the level of ophthalmologists, this may not always translate in practice, as they can lack generalizability [51]. In addition, the doctor-patient relationship in itself positively impacts patient care, as a systematic review of 25 randomized control trials found the doctor-patient relationship itself has a therapeutic effect on patients [64]. As the Hippocratic Oath states to “do no harm”, it is ideal to proceed with caution with AI programs, as they themselves have their own risks [63].

Rather than replacing jobs, AI in ophthalmology would be best utilized in conjunction with care provided by human ophthalmologists. AI programs may quickly synthesize large data sets allowing humans can spend more time with their patients. This method would ultimately enhance clinical efficiency while positively impacting patient care.

To truly visualize the impacts of AI on overall ophthalmic care, future research can focus on investigating overall patient outcomes with AI integration. Instead, many current AI studies explore the ability of AI programs to analyze data sets compared to human ophthalmologists without investigating overall patient outcomes [63]. Therefore, while AI is drastically improving, there needs to be further studies on the effect of AI on overall outcomes before raising concerns about its effect on jobs.

## Conclusions

AI has made significant progress in the fields of glaucoma, cornea, and oculoplastics. AI's integration into glaucoma management has significantly enhanced early detection and monitoring. AI advancements reduce interobserver variability, streamline diagnostic processes, and offer precise tools for glaucoma experts, ultimately improving patient care and outcomes. In the realm of corneal diseases, AI tools have shown remarkable promise in diagnosing and managing conditions such as keratitis, keratoconus, and diabetic keratoneuropathy. AI's role in oculoplastics is found in the effective diagnosis of orbital abscesses, thyroid ophthalmoplegia, and high-sensitivity ptosis screening. In addition, AI models assist in differentiating between various eyelid and orbital masses and also providing AI-driven measurements for surgical planning for oculoplastics cases.

Although there are limitations with AI utilization, such as loss of privacy, ethical concerns, and data set development, the rapidly changing nature of AI along with further studies in the field provides the opportunity to improve AI programs. With the augmentation of AI, it will likely find its place in patient care by working in conjunction with ophthalmologists to enhance the efficiency of data collection/analysis while allowing more time for doctor-patient interactions. As AI technologies continue to evolve, their applications in ophthalmology are expected to expand further, offering innovative solutions for the diagnosis, monitoring, treatment, and surgical outcomes of a wide range of eye conditions. The ongoing development and adoption of AI tools hold great promise for the future of ophthalmic care, ultimately leading to better patient outcomes and more efficient healthcare delivery.
